# Supplementary material for: Evaluation of cytological diagnostic accuracy for canine splenic neoplasms: An investigation in 78 cases using STARD guidelines
Source: PLoS One. 2019 Nov 7;14(11):e0224945. doi: 10.1371/journal.pone.0224945 (PMC6837434; doi:10.1371/journal.pone.0224945)
Supplement: Attachment — Submitted filename: PONE-D-19-20551R2 - Response to Reviewers.doc [file pone.0224945.s004.doc]

**REBUTTAL LETTER – Manuscript PONE-D-19-20551**

**RESPONSES TO REVIEWER 2**

1) “Remove "of" in line 57.”

Changed as suggested (current line 57).

2) “Lines 98-102 are not clear. Consider: "Application of STARD guidlines in the current study allowed cross-tabulation of cytological results (i.e. the index test) against those of histopathology (i.e. the reference standard) to generate sensitivity and specificity data. These data have not been included in previous studies and will be useful for future researchers comparing diagnostic methods for canine splenic neoplasms."”

Changed as suggested (current lines 98-103).

3) “Line 106, remove "a" before neoplasia (or say "a neoplasm")”

Changed as suggested (current line 107).

4) “Line 132, remove "considered as"”

Changed as suggested (current lines 132-133).

5) “Lines 218, 220, 222: add "the" before each category name (the True Positive (TP), etc.)”

Changed as suggested (current lines 219, 221, 223, 225).

6) “Line 305, add "The" before "Time interval"”

Changed as suggested (current line 306).

7) “Line 326: Either add an "and" before therefore, or change the comma to a semicolon.”

Changed as suggested (current line 327).

8) “Line 332: a dash is missing in "non-neoplastic."”

Changed as suggested (current line 333).

9) “Line 368: change "ratio" to "ratios"”

Changed as suggested (current line 369).

10) “Line 444: This is not clear. Consider "Moreover, identification of vascular tumors (angiomas and HES) among false negative cases is not surprising since the architecture of these tumors often leads to significant peripheral blood contamination in aspirates."”

Changed as suggested (current lines 445-447).

11) “Line 450: Change "by" to "of"”

Changed as suggested (current line 451).

12) “Line 451: Add "a" before low mitotic rate.”

Changed as suggested (current line 452).

13) “Line 462: Change the comma to a semicolon.”

Changed as suggested (current line 463).

14) “Line 471: Change "be considered also" to "also be considered."”

Changed as suggested (current line 472).

15) “Line 509: change "neoplasia, however high" to "neoplasia; however, high"”

Changed as suggested (current line 510).

Additional typos evidenced during the review were corrected as follows:

- Former line 45 (current line 45): double space preceding “Conversely” deleted.
- Former line 50 (current line 50): extra space preceding “Ultrasonographic” deleted.
- Former line 53 (current line 53): double space preceding “is” deleted.
- Former line 146 (current line 145): double space preceding “were” deleted.
- Former line 174 (current line 173): extra comma following “e.g.” deleted.
- Former line 182 (current line 181): double space preceding “specimens” deleted.
- Former line 190 (current line 189): extra space preceding “Neoplastic” deleted.
- Former line 347 (current line 346): double space preceding “Of” deleted.
- Former line 364 (current line 363): “was found, however severe marginal” reworded as “was found; however, severe marginal”.
- Former line 418 (current line 417): double space preceding “values” deleted.
- Former line 424 (current line 423): double space preceding “when” deleted.
- Former line 475 (current line 473): extra comma following “tumors” deleted.
- Former line 747 (current line 745): double space preceding “n/a” deleted.
